# A novel bacteriophage capable of efficiently lysing multi-drug-resistant *Klebsiella pneumoniae*

**DOI:** 10.3389/fmicb.2026.1877260

**Published:** 2026-08-05

**Authors:** Haipeng Zhang, Qianwen Cheng, Xiaohong Nie, Jinlian Gao, Yunxian Yang, Youhong Zhong, Liyuan Shi, Xiao Jin, Peng Wang

**Affiliations:** 1 Yunnan Provincial Key Laboratory of Natural Epidemic Diseases Prevention and Control Technology, Yunnan Institute of Endemic Diseases Prevention and Control, Dali, China; 2 Department of Epidemiology, Center for Global Health, School of Public Health, Nanjing Medical University, Jiangsu, Nanjing, China; 3 The Laboratory Department of Dali Bai Autonomous Prefecture Chinese Medicine Hospital, Yunnan, Dali, China

**Keywords:** biological characteristics, genome analysis, *Klebsiella pneumoniae*, multi-drug resistance, virulent bacteriophage

## Abstract

**Introduction:**

The increasing prevalence of carbapenem-resistant *Klebsiella pneumoniae* (CRKP) has posed a major challenge to clinical infection management. Phage therapy represents a promising alternative against multidrug-resistant bacterial infections; however, its application is limited by the scarcity of effective therapeutic phages. Therefore, the identification and characterization of novel phages are urgently needed.

**Methods:**

A novel Klebsiella pneumoniae phage was isolated and purified from hospital wastewater using the double-layer agar plate method with *K. pneumoniae* ATCC 700603 as the host strain. A total of 95 bacterial strains were used to evaluate the host range and lytic activity of the phage. The biological characteristics of the phage, including optimal multiplicity of infection (MOI), one-step growth curve, and stability under different environmental conditions (temperature, pH, ethanol, and ultraviolet exposure), were systematically investigated. Morphological characterization was performed by transmission electron microscopy. Whole-genome sequencing and bioinformatics analyses were conducted to determine genomic characteristics, taxonomic classification, and the presence of antibiotic resistance genes, virulence factors, or lysogeny-related genes to assess the therapeutic potential and biosafety of the phage.

**Results:**

The novel phage vB_KpnD_A2 exhibited potent lytic activity against multidrug-resistant Klebsiella pneumoniae strains. Host range analysis showed that vB_KpnD_A2 lysed 57.1% of carbapenem-resistant K. pneumoniae isolates and 63.6% of extended-spectrum β-lactamase-producing strains, while displaying specificity toward *K. pneumoniae*. The phage showed excellent biological properties, with an optimal MOI of 10^-^⁶, a latent period of 40 min, and a burst size of 8.6×10⁴PFU/cell. In addition, vB_KpnD_A2 maintained infectivity over a broad temperature range (4-70°C) and pH range (pH 2-13). Whole-genome analysis identified vB_KpnD_A2 as a strictly lytic Webervirus phage within the family Drexlerviridae, without detectable antibiotic resistance genes, virulence factors, or lysogeny-associated genes, supporting its therapeutic potential and biosafety.

**Discussion:**

The newly characterized phage vB_KpnD_A2 exhibited potent lytic activity against selected multidrug-resistant Klebsiella pneumoniae isolates, favorable genomic safety, and good environmental stability. These findings support its potential as a therapeutic phage candidate for CRKP control and expand anti-Klebsiella phage resources for future phage-based interventions against antimicrobial-resistant bacterial infections.

## 1 Introduction

*Klebsiella pneumoniae* (KP) is a Gram-negative bacterium. As a conditionally pathogenic bacterium widely present in nature, it has been included in the “ESKAPE” group (*Enterococcus faecalis*, *Staphylococcus aureus*, *K. pneumoniae*, *Acinetobacter baumannii*, *Pseudomonas aeruginosa*, and *Enterobacteriaceae*; Lu et al., 2022; Mulani et al., 2022). When the immune system of the human body or animals is weakened or the internal bacterial flora is imbalanced, *K. pneumoniae* can cause a series of infections, including pneumonia, soft tissue and surgical wound infections, urinary tract infections, sepsis, etc., with significant clinical incidence and mortality rates (Dong et al., 2022). In recent years, the prevalence of *K. pneumoniae* has been on a continuous rise with a multidrug-resistant trend, and it is estimated that this pathogen could cause 10 million deaths worldwide by 2050 (Peng et al., 2025). For the management of such infections, high reliance is placed on the combination therapy of antibiotics including colistin and aminoglycosides in clinical settings, though these medications pose toxicity risks and demonstrate restricted efficacy (Kou et al., 2024). Especially, carbapenem-resistant hypervirulent *K. pneumoniae* (CR-hvKP) that has acquired resistance to these antibiotics and emerged recently has aggravated the predicament of clinical therapy, with an urgent requirement for the development of novel antimicrobial agents and alternative therapeutic strategies (Fang et al., 2023).

Bacteriophages are a type of virus capable of specifically infecting bacteria; they are widely present in the natural environment and exhibit remarkably rich genetic diversity (Senhaji-Kacha et al., 2024). Due to their high host specificity and non-toxicity to eukaryotic cells, they have garnered significant attention in the research community (Chen et al., 2023). A major advantage of bacteriophages relative to traditional antibiotics is that they can co-evolve with bacteria; when antibiotic resistance mechanisms are developed by bacteria, such resistance can be overcome by phages via adaptation and evolution (Saqr et al., 2024). And they are regarded as the most promising ultimate therapeutic modality for complex and intractable bacterial infections (Liu K. et al., 2022). Bacteriophages are classified into two types based on their infection mechanism: virulent and lysogenic. Among them, virulent bacteriophages infect and lyse the bacterial host, and can be used for treating pathogenic bacterial infections (Li et al., 2021). Nowadays, single bacteriophages or mixtures of several lysogenic bacteriophages have been successfully applied in personalized treatment (Gorodnichev et al., 2023). However, the application of phage therapy also faces numerous challenges, such as limited host range, phage resistance and lysogenic conversion, further underscoring the critical importance of establishing systematic phage libraries (Sadeqi et al., 2023).

In this context, the continuous discovery and characterization of novel virulent bacteriophages not only facilitates the construction of biological resource banks, but also serves as the foundation for preparing cocktail therapies to combat phage resistance. Consequently, this study focuses on the bacteriophage vB_KpnD_A2, isolated from sewage and targeting multidrug-resistant *K. pneumoniae*, to analyze its host range, lytic capacity, stability, and whole-genome sequence, thereby providing scientific evidence for its potential as a candidate for phage therapy.

## 2 Materials and methods

### 2.1 Bacterial strains

**Figure 1:**
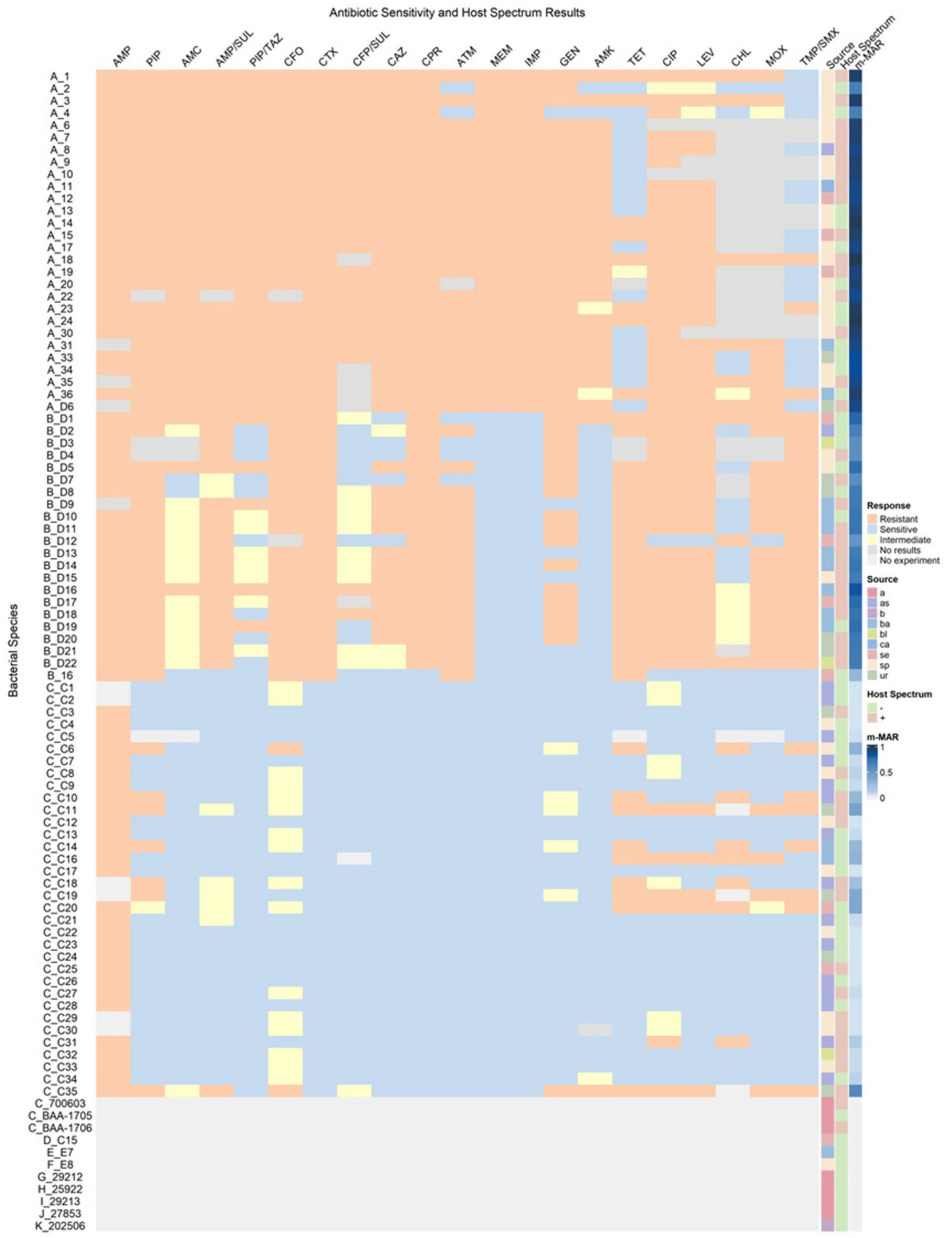
Lysis spectrum and antibiotic susceptibility results of phage vB_KpnD_A2. Strains: (A) carbapenem-resistant *Klebsiella pneumoniae* (CRKP); (B) extended-spectrum β-lactamase (ESBL)-producing *K. pneumoniae*; (C) other unclassified *K. pneumoniae*; (D) *Klebsiella oxytoca*; (E) *Klebsiella ozaenae*; (F) *Klebsiella aerogenes*; (G) *Enterococcus faecium*; (H) *Escherichia coli*; (I) *Staphylococcus aureus*; (J) *Pseudomonas aeruginosa*; (K) *Yersinia enterocolitica*. Origin: (a) standard strains from Yunnan Clinical Testing Center; (b) inter-laboratory quality control strains from the National Health Commission Clinical Testing Center. Specimen types: sp., sputum; as, aspiration fluid; bl, blood; ur, urine; se, secretion; ba, bronchoalveolar lavage fluid; ca, catheter tip.

We used 87 strains of *K. pneumoniae*, including three standard strains (ATCC 700603, ATCC BAA-1705, ATCC BAA-1706), 28 carbapenem-resistant *K. pneumoniae* strains, 22 extended-spectrum β-lactamase-producing *K. pneumoniae* strains, and 37 unclassified other *K. pneumoniae* strains. Additionally, we used *acid-producing Klebsiella* C15, *malodorous nose Klebsiella* E7, *gas-producing Klebsiella* E8, *E. faecalis* ATCC 29212, *Escherichia coli* ATCC 25922, *S. aureus* ATCC 29213, *P. aeruginosa* ATCC 27853, and *Yersinia enterocolitica* 202506. A total of 95 bacterial strains were used for the determination of the host range (Figure 1).

### 2.2 Isolation and identification of bacteriophages

Bacteriophage vB_KpnD_A2 was isolated and purified from water samples collected at the sewage treatment facility of Dali Prefecture Hospital of Traditional Chinese Medicine, using ATCC700603 as the host bacterium and employing the double-layer plaque assay method (Chhibber et al., 2018). Bacteria were cultured in Luria-Bertani (LB) medium, either on solid agar plates containing 1.5% agar or in liquid broth. Cultures were incubated at 37 °C with shaking at 220 rpm/min.

### 2.3 Electron microscopic morphology

Purified bacteriophage suspensions were subjected to morphological analysis using a JEM-1400Flash transmission electron microscope (JEOL, Japan) at the Electron Microscopy Laboratory of the Scientific Research Center, Kunming Medical University. For negative staining, a droplet of the suspension was applied to a carbon-coated copper grid and allowed to adsorb for 2 min. Excess liquid was subsequently removed by gentle blotting with filter paper. The grid was then stained with 2% (w/v) phosphotungstic acid (pH 6.5) for 1 min and air-dried at ambient temperature. Imaging was performed under an acceleration voltage of 100 kV using the instrument’s integrated Flash CMOS camera system (Terzian et al., 2021).

### 2.4 Antibiotic sensitivity test

Antimicrobial susceptibility testing of clinical isolates of *K. pneumoniae* was performed against a panel of antibiotics, including but not limited to ampicillin, piperacillin, amoxicillin/clavulanic acid, ampicillin/sulbactam, piperacillin/tazobactam, cefazolin, cefotaxime, cefoperazone/sulbactam, ceftazidime, cefepime, aztreonam, meropenem, imipenem, gentamicin, amikacin, tetracycline, ciprofloxacin, levofloxacin, chloramphenicol, moxifloxacin, and trimethoprim/sulfamethoxazole (TMP/SMX). Testing was conducted using the BD Phoenix™ M50 automated system for microbiology identification and susceptibility analysis (Tüzemen et al., 2024).

### 2.5 Determination of the phage host range

The lytic activity of bacteriophages was assessed by applying 20 μL of phage suspension onto bacterial colony plates. Following overnight incubation, the formation of clear plaques indicated successful bacterial lysis (+), whereas the absence of plaques denoted no lytic activity against the target bacteria (−) (Huang et al., 2025). All experiments were conducted in triplicate.

### 2.6 Optimal multiplicity of infection (MOI)

The optimal MOI was determined with a modified protocol based on established methodologies (Liu B. et al., 2022). Briefly, phage suspensions and host bacterial cultures were mixed in equal volumes at MOI gradients ranging from 10^−8^ to 10^1^. The mixtures were incubated overnight at 37 °C with continuous shaking. Phage titers were subsequently quantified using the standard double-layer agar plate technique. The MOI yielding the maximal phage titer was identified as optimal. All experiments were conducted in triplicate.

### 2.7 One-step growth curve

Phage particles and host bacteria were mixed at the optimal MOI ratio in equal volumes, following the methodology established by Wang J. R. et al. (2019). The mixture was incubated at 37 °C in a water bath for 15 min, followed by centrifugation and supernatant removal. The pellet was washed twice with liquid LB medium and resuspended. Subsequently, the suspension underwent continuous incubation at 37 °C in a water bath. At designated time points (0, 5, 10, 20, 30, 40, 50, 60, 70, 75, 80, 85, 90, 95, 100, 110, and 120 min), aliquots were collected and centrifuged. From the supernatant, 100 μL was subjected to serial dilution, and phage titers were determined to construct a one-step growth curve. Burst size was calculated by dividing the phage titer at the end of the burst phase by the initial host bacterial concentration (Wang R. et al., 2019). All experiments were conducted in triplicate.

### 2.8 Temperature and pH stability

To assess the thermal stability of bacteriophages, bacteriophage suspensions were incubated in a constant-temperature water bath at 4, 21, 28, 37, 50, 60, 70, and 80 °C for 1 h. Subsequently, 100 μL of the bacteriophage suspension was subjected to serial dilution, and the titer was determined to evaluate stability. For pH stability assessment, 100 μL of bacteriophage suspension was mixed with an equal volume of PBS buffer adjusted to pH values ranging from 1 to 14. The mixture was incubated at 37 °C for 1 h, followed by serial dilution and titer measurement. All experiments were conducted in triplicate.

### 2.9 Ethanol and ultraviolet sensitivity

Bacteriophage suspensions were mixed with ethanol to achieve final concentrations of 10, 25, 50, 75, and 90%, followed by incubation at 37 °C in a water bath. Aliquots of 100 μL from each ethanol concentration mixture were collected at 30 min and 60 min for analysis. Phage activity (titer) was assessed using a spot-drop assay. To assess ultraviolet (UV) sensitivity, 4 mL of bacteriophage suspension was placed at the center of a biological safety cabinet and exposed to UV light. Aliquots of 100 μL were collected at 3-min intervals from 0 to 40 min for subsequent analysis. Phage activity (titer) was assessed using a spot-drop assay. All experiments were conducted in triplicate.

### 2.10 Whole genome sequencing and bioinformatics analysis of bacteriophages

The extraction of phage DNA was carried out using the balanced phenol–chloroform–isoamyl alcohol method described by Sambrook and Russell (2001), and then the samples were sent for sequencing. The CGview Server was used to analyze overall genomic features; tRNAscan-SE was employed for tRNA prediction; the Comprehensive Antibiotic Resistance Database (CARD) and Virulence Factor Database (VFDB) were utilized to screen for antibiotic resistance genes and virulence factors, respectively; and PhageLeads was utilized for predicting lysogeny-related genes. Gene function prediction was performed using the RAST online system, with results annotated by comparison against the NCBI BLAST database and with reference to VIBRANT 1.2.1. The genomic map was generated using CLC Genomics Workbench 12.02. JSpeciesWS^1^ was applied to calculate the Average Nucleotide Identity (ANI) and coverage between phages, and the online tool taxMyPhage^2^ was used to predict the taxonomic classification of the phage. Furthermore, the amino acid sequences of the phage terminase large subunit and major capsid protein were subjected to BLASTp analysis against the NCBI database. The 25 reference phages with the highest similarity were selected for multiple sequence alignment. Multiple sequence alignment was performed using the Clustal W program in MEGA 7.0, and a phylogenetic tree was constructed based on the Neighbor-Joining method, with branch stability evaluated by 1,000 bootstrap replicates. From the reference phages showing high homology, the five with the highest similarity were selected, and a synteny map was generated using Easyfig 2.2.

## 3 Results

### 3.1 Morphology and titer of bacteriophage vB_KpnD_A2

**Figure 2:**
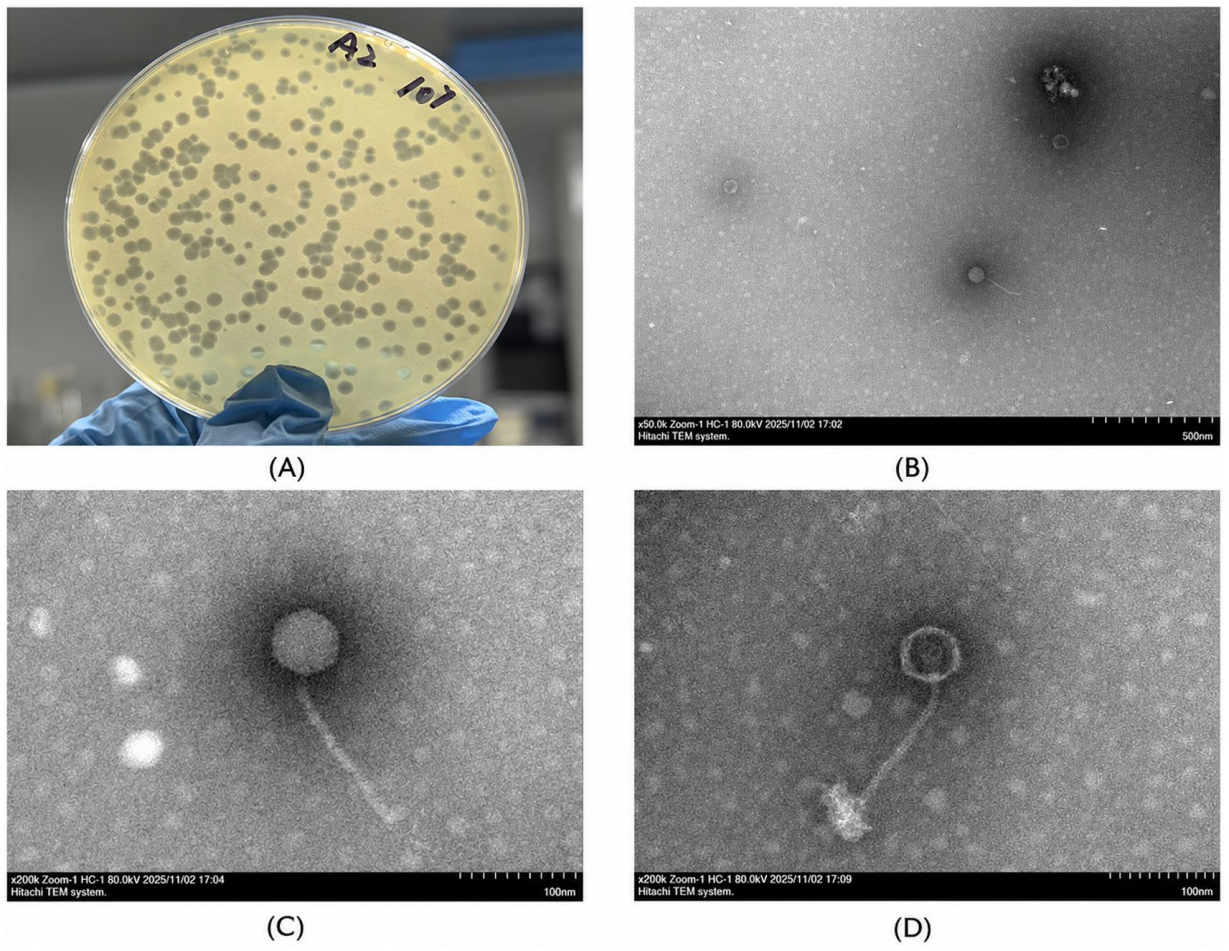
The morphology of phage vB_KpnD_A2. Phage vB_KpnD_A2 plaque **(A)**, TEM image of phage vB_KpnD_A2 **(B–D)**, with a scale of 100 nm.

A *K. pneumoniae*-targeting bacteriophage, designated vB_KpnD_A2, was successfully isolated from hospital wastewater. This phage formed clear, circular plaques (diameter almost is 1–4 mm) with distinct halos on double-layer agar plates (Figure 2A). Transmission electron microscopy (TEM) analysis revealed virions with a tad-pole-like morphology, comprising an icosahedral head (diameter: 100 ± 5.0 nm) and a tail measuring 190 ± 10.0 nm in length (Figures 2B–D). Following multiple purification and amplification cycles, the phage achieved an average titer of 6 × 10^13^ PFU/mL, markedly exceeding titers re-ported for most documented *K. pneumoniae* phages.

### 3.2 Antibiotic sensitivity results

We conducted drug sensitivity tests for 21 kinds of antibacterial drugs on 84 clinically isolated *K. pneumoniae* strains. The results showed that the m-MAR index of 70.2% (59/84) of *K. pneumoniae* isolates was higher than 0.2, among which 59.5% (50/84) of the isolates reached a level above 0.5, and 22.2% (17/84) of the isolates reached a level above 0.9. Further analysis revealed that the m-MAR index of all CRKPs was greater than 0.85. Except for remaining sensitive to tetracycline and bactrim, they showed extensive resistance to the vast majority of the tested drugs (Figure 1). In contrast, the overall drug resistance of extended-spectrum β-lactamase (ESBL) producing strains was relatively low, but they were still insensitive to 66.7% of the tested drugs. All three standard strains of *K. pneumoniae* were not resistant.

### 3.3 Host profile results

The host lysis profile analysis results of the bacteriophage vB_KpnD_A2 (Figure 1) showed that the lysis rate of this bacteriophage against 28 carbapenem-resistant *K. pneumoniae* (CRKP) was 57.1% (16/28), and the lysis rate against 22 strains producing extended-spectrum β-lactamase (ESBL) was 63.6% (14/22). The lysis rate against the other 40 strains of *K. pneumoniae* was 45.0% (18/40). Further analysis revealed that vB_KpnD_A2 still had lysis ability against 62.0% (31/50) strains with an m-MAR index higher than 0.2. In addition, vB_KpnD_A2 exhibits relatively strict host specificity and does not lyse strains other than *K. pneumoniae*.

### 3.4 Optimal multiplicity of infection

**Figure 3:**
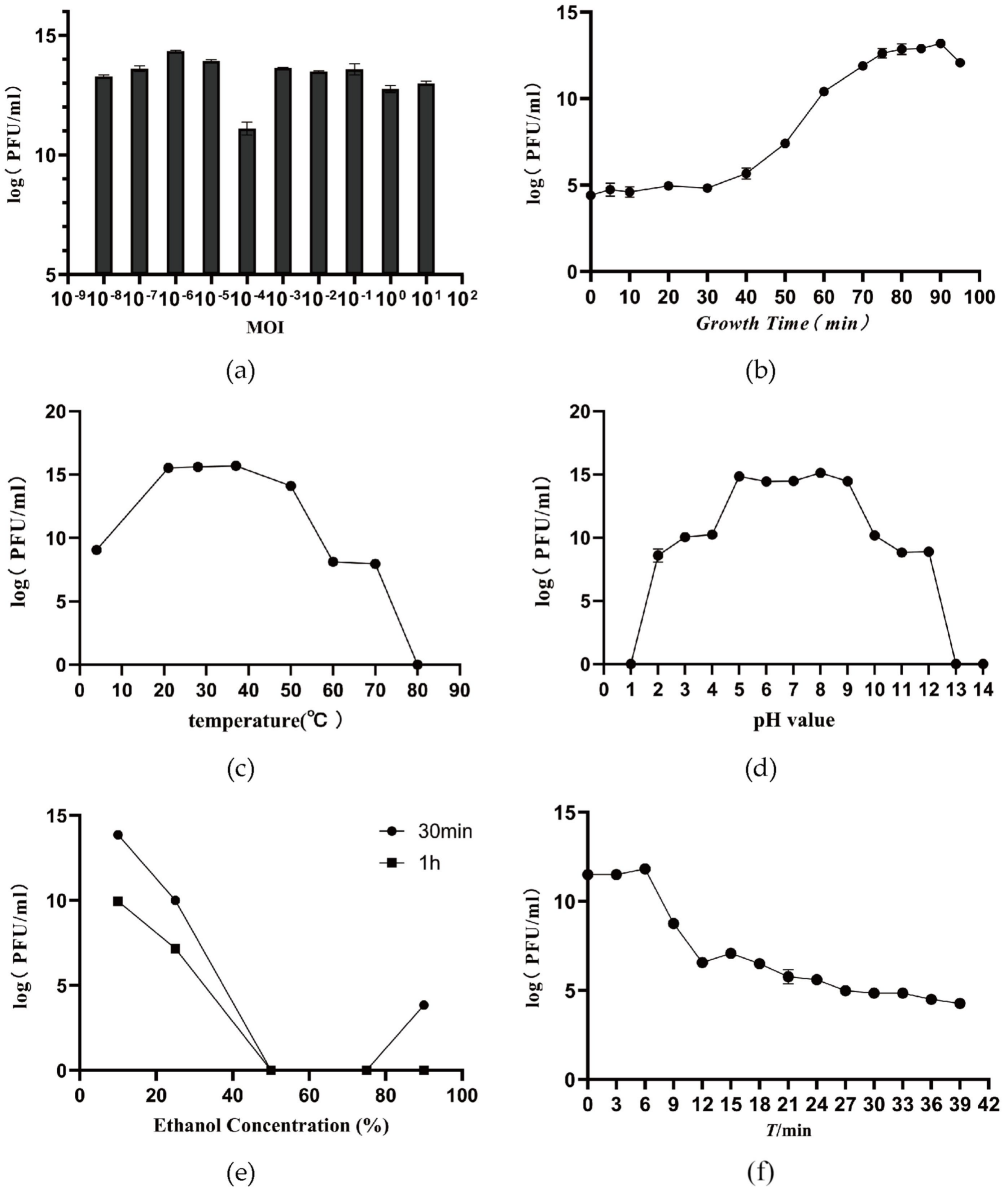
Biological characteristics of bacteriophage vB_KpnD_A2. Optimal MOI **(a)**, one-step growth curve **(b)**, thermal stability **(c)**, acid–base inactivation effect **(d)**, ethanol sensitivity **(e)**, and ultraviolet tolerance **(f)** of vB_KpnD_A2 genome analysis of phage vB_KpnD_A2.

The optimal multiplicity of infection (MOI) for phage vB_KpnD_A2 is 0.000001. Under this optimal MOI, the progeny phage yield reaches 2 × 10^14^ PFU/mL (Figure 3a).

### 3.5 One-step growth curve

The one-step growth curve of phage vB_KpnD_A2 (Figure 3b) demonstrates a latent period of 40 min and a lysis duration of 35 min. The burst size is 8.6 × 10^4^ PFU/cell, indicating that upon lysis, a single infected host bacterium releases 8.6 × 10^4^ progeny phages.

### 3.6 Temperature and pH stability

Thermal stability assays reveal that phage vB_KpnD_A2 retains activity when incubated between 4 °C and 70 °C. However, the phage becomes completely inactivated at 80 °C (Figure 3c). Under identical incubation conditions, phage vB_KpnD_A2 maintains infectivity across an exceptionally broad pH range, from pH 2 to pH 13 (Figure 3d), highlighting its robust environmental resilience.

### 3.7 Ethanol and UV sensitivity

Ethanol sensitivity tests show that treatment with 50% or 75% ethanol for 30 min leads to complete inactivation of phage vB_KpnD_A2 (Figure 3e). Ultraviolet (UV) radiation sensitivity assays indicate a progressive decline in phage titer with increasing exposure time (Figure 3f). Despite continuous reduction in viability, the phage retains detectable activity even after 40 min of UV exposure, suggesting partial resistance to UV-induced damage. This indicates that while UV light effectively reduces phage infectivity over time, it does not immediately abolish all viral function under the tested conditions.

### 3.8 General genomic characteristics of phage vB_KpnD_A2

The genome of phage vB_KpnD_A2 is a linear double-stranded DNA molecule of 47,683 bp with a GC content of 50.9% (Supplementary Figure S1). Taxonomic assignment via taxMyPhage identified vB_KpnD_A2 as a member of the genus Webervirus within the family Drexlerviridae. The GenBank accession number is PV131042.1. Further genomic safety assessment revealed that this phage lacks tRNA and lysogeny-related genes, indicating it is strictly lytic. Moreover, no antibiotic resistance genes or virulence factors were detected, confirming its favorable safety profile for potential clinical applications.

**Figure 4:**
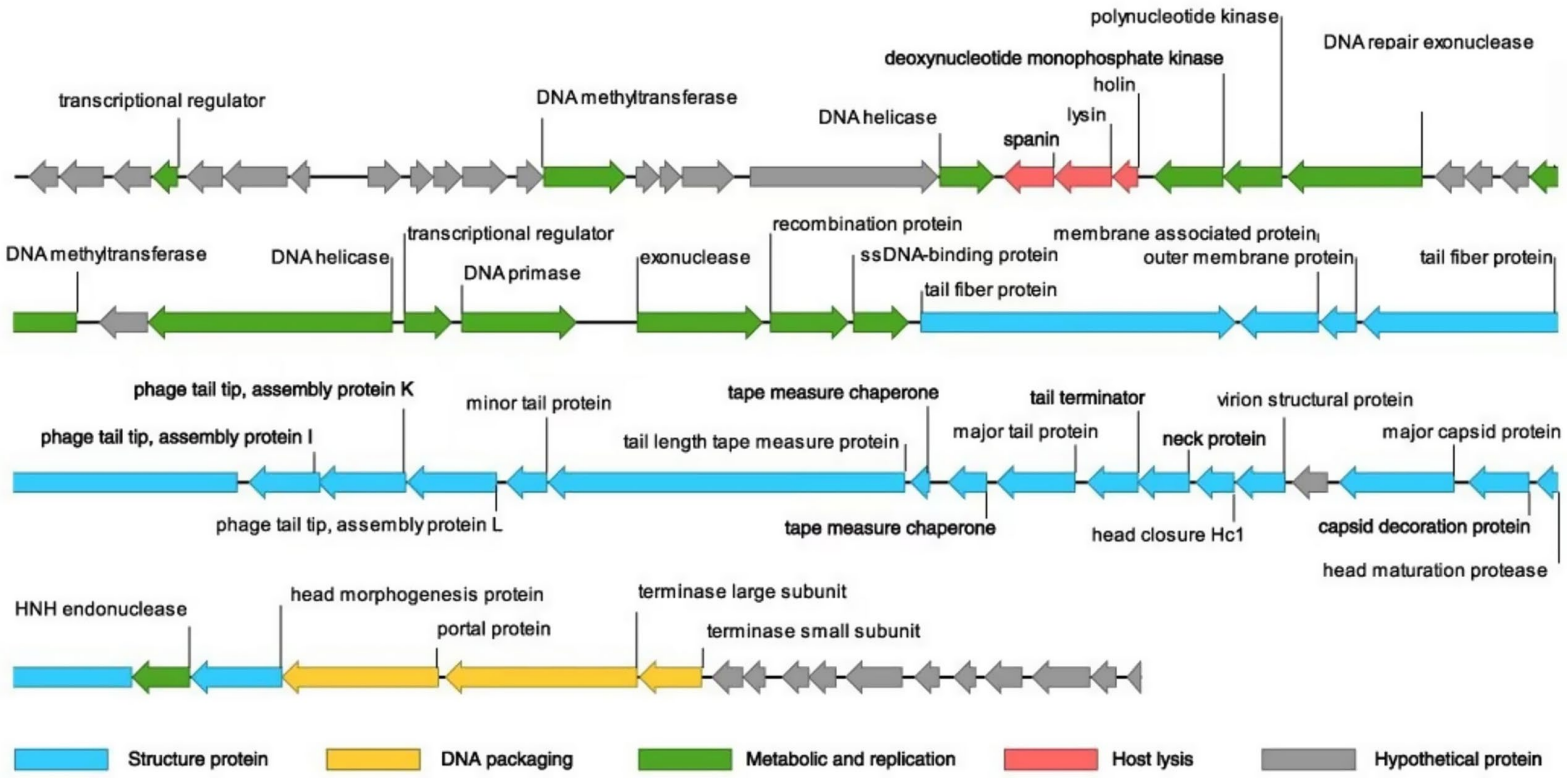
Genomic map of bacteriophage vB_KpnD_A2. Color-coded by functional category (e.g., structural proteins, DNA packaging, metabolism and replication, host lysis).

Based on RAST and BLASTp analyses, vB_KpnD_A2 contains 71 open reading frames (ORFs). RAST predicted 32 coding sequences (CDSs), while the remaining 39 ORFs were annotated as “hypothetical proteins” (Figure 4). The 32 CDSs were functionally categorized into four modules: phage structural proteins, replication and metabolism, assembly regulation, and lysis functions, as detailed in the Supplementary material. Among them, 14 CDSs are related to phage structure, such as tail fiber protein, phage tail tip assembly protein I, tail terminator, and head morphogenesis protein; 13 CDSs are involved in phage replication and metabolism, including DNA methyltransferase, polynucleotide kinase, recombination protein, and DNA repair exonuclease; six CDSs are associated with DNA packaging, such as head closure protein Hc1, portal protein, and terminase large subunit; and three CDSs—spanin, lysin, and holin—are involved in host cell lysis.

### 3.9 Average nucleotide identity (ANI) analysis

**Figure 5:**
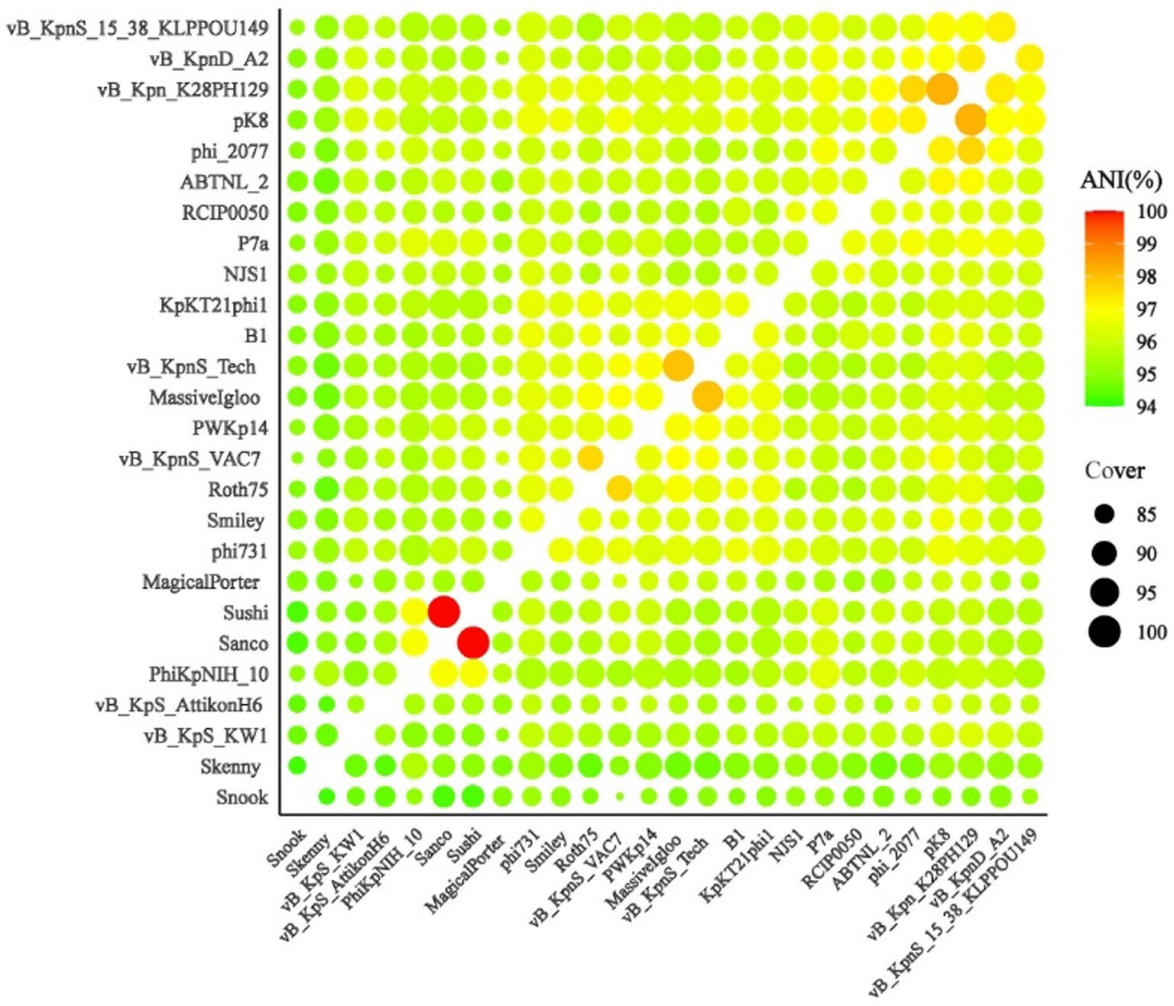
Heatmap of bacteriophage vB_KpnD_A2 (showing ANI and coverage with reference phages).

ANI analysis results (Figure 5) showed that vB_KpnD_A2 shares 97%–99% ANI with vB_KpnS_15_38_KLPPOU149, vB_Kpn_K28PH129, pK8, phi_2077, ABTNL-2, with coverage > 95%. According to the species demarcation criterion of ANI ≥ 95% (Coutinho et al., 2023), and the next-generation phage classification system based on genomic composition (Turner et al., 2025). vB_KpnD_A2 can be classified as the same species as these phages and is a novel isolate of this species.

### 3.10 Comparative genomic sequence analysis

**Figure 6:**
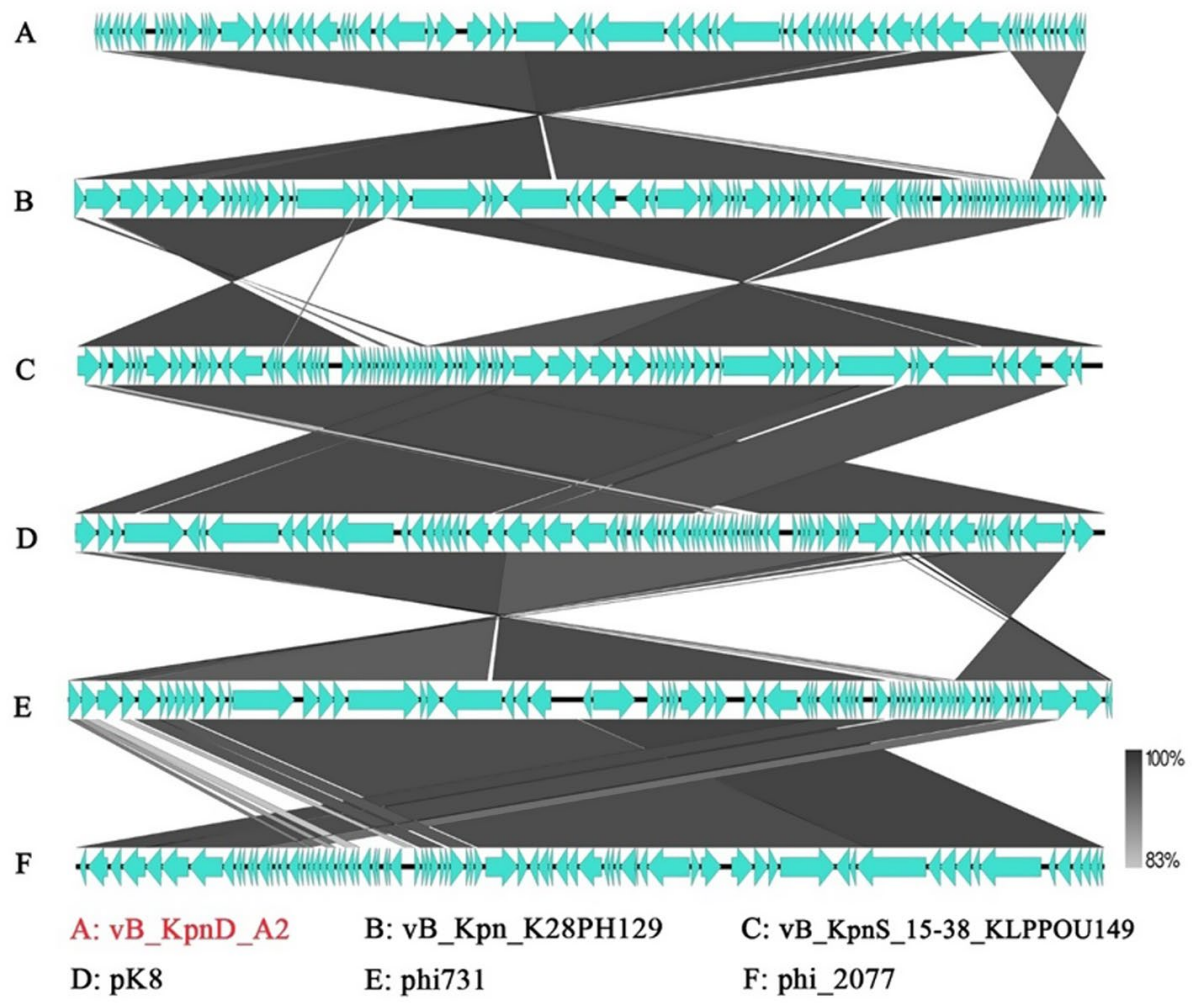
Linear alignment map of the vB_KpnD_A2 genome with reference phages. (A: vB_KpnD_A2; B: vB_Kpn_K28PH129; C: vB_KpnS_15-38_KLPPOU149; D: pK8; E: phi731; F: phi_2077).

BLASTn analysis revealed that phage vB_KpnD_A2 exhibits high homology with several Klebsiella phages, showing the highest similarity (97.46, 97% coverage) with vB_KpnS_15_38_KLPPOU149 (Table 1). Further Easyfig synteny analysis indicated that vB_KpnD_A2 shares a similar overall genomic module organization with these five reference phages but displays some differences in gene arrangement (Figure 6).

**Table 1:** The top five phages with the highest genomic similarity to bacteriophage vB_KpnD_A2.

Phage	Region (year)	Coverage (%)	Identity (%)
*Klebsiella* phage vB_Kpn_K28PH129	Spain (2023)	98	97.12
*Klebsiella* phage vB_KpnS_15_38_KLPPOU149	USA (2023)	97	97.46
*Klebsiella* phage pK8	Australia (2022)	98	97.19
*Klebsiella* phage phi731	Hungary (2023)	98	97.07
*Klebsiella* phage phi_2077	South Korea (2023)	95	96.87

### 3.11 Phylogenetic analysis

**Figure 7:**
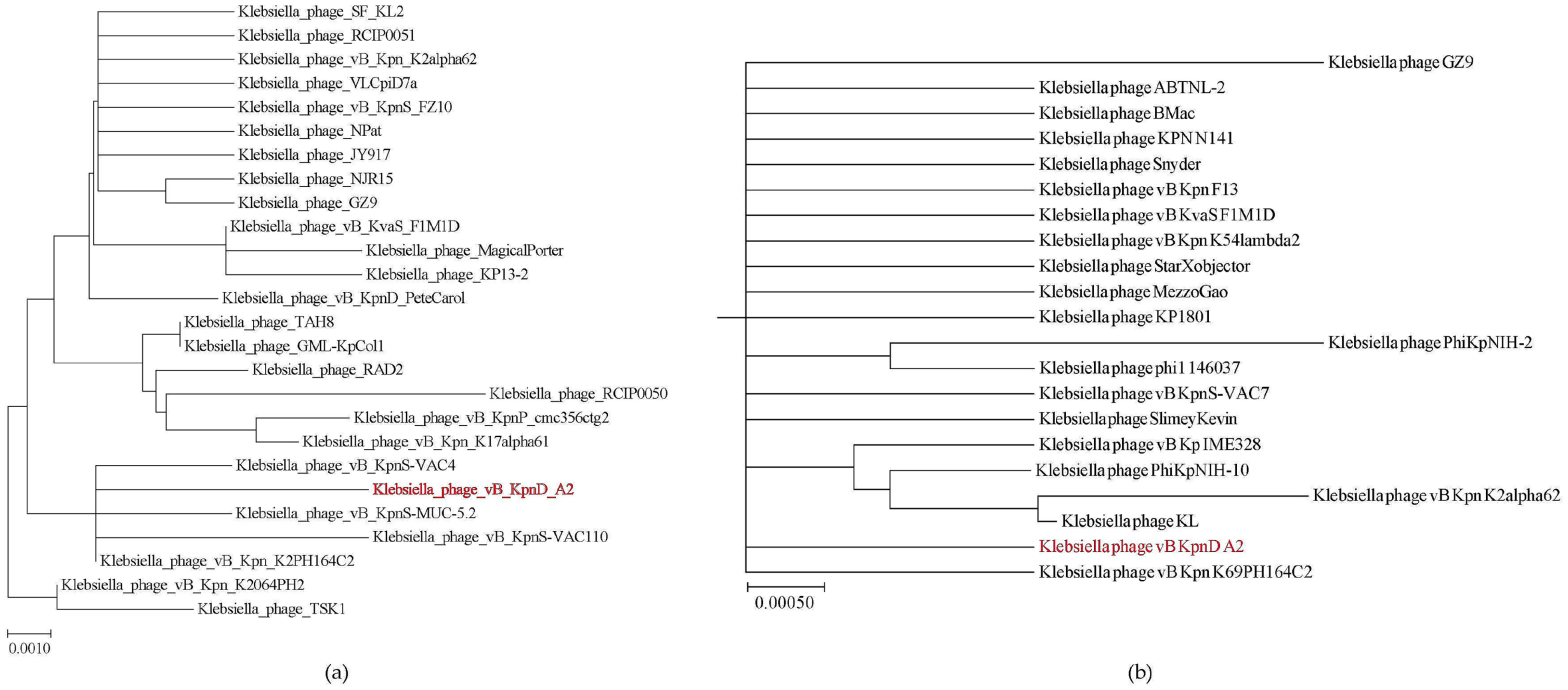
Phylogenetic trees constructed based on **(a)** major capsid protein and **(b)** terminase large subunit. The trees were generated using the neighbor-joining method with 1,000 bootstrap replicates.

The 25 reference strains with the highest similarity were selected to infer the evolutionary relationship of vB_KpnD_A2 with other phages by constructing phylogenetic trees based on the major capsid protein (Figure 7a) and the terminase large subunit (Figure 7b), as these proteins are conserved. The results showed that vB_KpnD_A2 clusters within the same branch as vB_KpnS-VAC4, vB_KpnS-MUC-5.2, vB_KpnS-VAC110, vB_Kpn_K69PH164C2, indicating a close evolutionary relationship. Notably, in the terminase large subunit tree, vB_KpnD_A2 occupies a relatively distinct branch, suggesting some variation in this gene. Integrating the results from both trees, vB_KpnD_A2 belongs to the genus Webervirus within the family Drexlerviridae, consistent with the taxMyPhage classification. Combined with ANI and genomic synteny analyses, these findings further support that vB_KpnD_A2 is a novel isolate within the same species of the genus Webervirus.

## 4 Discussion

Hospital wastewater, long-term exposed to high concentrations of antibiotics, serves as a reservoir for multidrug-resistant (MDR) bacteria. In this highly selective environment, phages capable of lysing drug-resistant *K. pneumoniae* are more readily isolated (Mirza et al., 2025). Moreover, long-term coexistence and co-evolution of phages and host bacteria in wastewater promote genetic variation in phages, equipping them with the ability to infect various phenotypic bacterial pathogens (Yuan et al., 2018). The lytic spectrum assay of phage vB_KpnD_A2, isolated from wastewater in this study, demonstrated that its lytic spectrum is restricted to *K. pneumoniae*, indicating high specificity and a significantly reduced risk of impacting normal flora. In this study, antimicrobial susceptibility testing of 84 clinical *K. pneumoniae* isolates revealed that over half exhibited a multiple antibiotic resistance (MAR) index greater than 0.5. vB_KpnD_A2 effectively lysed 60.2% of these (MDR) strains, suggesting its clear potential for development in treating infections caused by (MDR) bacteria.

Phages with high application value typically possess high burst size, good stability, and encode capsule depolymerases (Dawson et al., 2025). vB_KpnD_A2 forms clear plaques 1–4 mm in diameter with obvious halos on the lawn of host strain ATCC700603. This characteristic suggests the possible presence of functional depolymerases (Liang et al., 2022), which may penetrate host biofilms and enhance the lytic efficacy against resistant bacteria (Cui et al., 2023). A lower optimal multiplicity of infection (MOI) indicates stronger infectivity. The optimal MOI of vB_KpnD_A2 is 0.000001, with a latent period of 40 min, a rise period of 35 min, and a burst size of 8.6 × 10^4^ PFU/cell, which is considerably higher than that of typical *K. pneumoniae* phages. Its optimal MOI and one-step growth curve demonstrate the phage’s highly efficient ability to infect and lyse *K. pneumoniae*, showing potential for clinical application.

Good thermal and pH stability are key physicochemical properties for phages to withstand various stress conditions during production, storage, and complex application environments, directly determining the breadth and feasibility of their practical use (Kowalska et al., 2020). vB_KpnD_A2 not only exhibits significant tolerance to high temperature (70 °C) and UV irradiation but also possesses an extremely wide pH adaptation range (pH 2–12), covering conditions from strong acid to strong alkali, demonstrating its robust survivability in harsh environments. Regarding drug delivery routes, oral administration of phages is widely recognized for its safety and convenience, particularly suitable for treating gastrointestinal and extraintestinal infections (Singh et al., 2024). Based on its excellent environmental stability, vB_KpnD_A2 holds potential for clinical translation in oral, topical, systemic, and invasive surgical applications (Mirza et al., 2025), promising to provide a reliable biological agent for the prevention and control of resistant bacteria.

Genomic analysis revealed that ORFs 19–21 constitute the phage lysis module, annotated as spanin, lysin, and holin, respectively. These three proteins cooperate to accomplish bacterial lysis: holin and lysin work together to disrupt the cell wall; subsequently, spanin forms a complex spanning the inner and outer membranes to promote their fusion, ultimately leading to bacterial lysis (Samir, 2024). Based on NCBI BLASTp comparisons, the amino acid sequence of the holin shares 98.59% similarity with other phage sequences, differing by only one amino acid. The lysin shares 96.88%–99.38% similarity with the top 30 matched sequences, differing by 1–5 amino acids. The spanin also shares over 98.58% similarity with the top 30 matches, all of which are Klebsiella phages, reflecting the conservation of lysis proteins among phages of the same species.

Phylogenetic analysis suggests that environmental-genetic interactions may have led to alterations in the terminase large subunit of this phage. Its gene arrangement differs somewhat from other highly similar phages, confirming it as a novel phage within the genus Webervirus, family Drexlerviridae. Studies indicate that virulence genes in pathogenic bacteria often originate from integrated prophages. The absence of virulence genes in this phage ensures that its therapeutic use will not lead to the emergence of new strains carrying specific virulence determinants and effectively prevents the production of directly host-toxic metabolites during treatment. Furthermore, the lack of antibiotic resistance genes in its genome significantly reduces the risk of horizontal gene transfer, meaning it will not confer resistance to host bacteria during therapy, fundamentally avoiding the emergence of resistant strains and providing crucial safety assurance for its clinical application.

## 5 Conclusion

Using the standard *K. pneumoniae* strain ATCC700603 as the host, this study successfully isolated a novel long-tailed lytic phage, vB_KpnD_A2, from hospital wastewater. Its nucleic acid is double-stranded DNA, and it is classified as a novel member of the genus Webervirus, family Drexlerviridae, meeting biosafety standards for phage therapy. It can lyse multidrug-resistant strains, has an extremely low optimal MOI (0.000001), and a very high burst size (8.6 × 104 PFU/cell), which could reduce production costs and improve economic efficiency in future clinical applications. It maintains high lytic efficacy in complex *in vitro* and *in vivo* environments, demonstrating strong environmental adaptability. These characteristics provide a reliable safety foundation for its application in complex clinical settings and underscore its potential as an emerging biological antimicrobial agent for the prevention and control of infections associated with multidrug-resistant *K. pneumoniae*.

## Data Availability

The datasets presented in this study can be found in online repositories. The names of the repository/repositories and accession number(s) can be found in the article/Supplementary material.

## Footnotes

1
https://jspecies.ribohost.com/

2
https://phagecompass.ku.dk/
